# Prevalence of cardiometabolic risk factors according to urbanization level, gender and age, in apparently healthy adults living in Gabon, Central Africa

**DOI:** 10.1371/journal.pone.0285907

**Published:** 2024-04-05

**Authors:** Mérédith Flore Ada Mengome, Héléna Noéline Kono, Elsa Ayo Bivigou, Noé Patrick M’bondoukwe, Jacques-Mari Ndong Ngomo, Bridy Moutombi Ditombi, Bedrich Pongui Ngondza, Cyrille Bisseye, Denise Patricia Mawili-Mboumba, Marielle Karine Bouyou Akotet

**Affiliations:** 1 Département de Parasitologie-Mycologie, Université des Sciences de la Santé (USS), Libreville, Gabon; 2 Laboratoire de Biologie Moléculaire et Cellulaire (LABMC), Université des Sciences et Techniques de Masuku (USTM), Franceville, Gabon; 3 Faculté de Médecine, Département de Médecine, Université des Sciences de la Santé, Libreville, Gabon; Navrongo Health Research Centre, GHANA

## Abstract

**Background:**

The prevalence of cardiometabolic risk factors (CMRFs) is increasing in sub-Saharan Africa and represents a serious public health issue. Accurate data are required to implement adapted prevention programs and healthcare strategies. Thus, the aim of this study was to estimate the prevalence rates of CMRFs according to the level of urbanization, age and gender in Gabon.

**Methods:**

A cross-sectional study was conducted in northern (Bitam), western coast (Libreville, Melen) and southeast (Koulamoutou) areas of Gabon using the World Health Organization’s (WHO) stepwise approach for the surveillance of chronic disease risk factors. Participants over 18 years of age, without known underlying disease, living in rural and urban areas of Gabon were included. Sociodemographic, biological, and behavioral data were collected. Univariate and multivariate analysis were used to identify the CMRFs.

**Results:**

Of the 978 participants, 499 lived in urban and 479 in rural areas. Their median age was 38[28–50] years. Tobacco (26.1% vs 6.2%; p < 0.01) and excessive alcohol consumption (19.4% vs 9.6%; p < 0.01) predominated in rural than in urban areas, respectively. Urban dwellers had more often insufficient physical activity than rural people (29.5% vs 16.3%; p < 0.01). In total, 79.9% of participants aged under 54 years had a high blood pressure;10.6% of the younger participants had pre-hypertension. Metabolic syndrome was more frequent in women (21.7%) than in men (10.0%) (p < 0.01); 6.4% of men and 2.5% of women had a high Framingham score (p = 0.03). Finally, 54.0% of the participants had three or four CMRFs. The multivariate analysis showed that men were more likely to be smokers and to be at risk of pre-hypertension or high blood pressure (p < 0.01). Women were more likely to be obese or to have a metabolic syndrome (p < 0.01). Living in urban areas was also a risk factor for hypertension, diabetes, metabolic syndrome and high LDL cholesterol level.

**Conclusion:**

The prevalence of CMRFs was high in the study population. Disparities were observed according to urban and rural areas, gender and age. National prevention and healthcare strategies for cardiometabolic diseases in Gabon should consider these observed differences.

## Introduction

Cardiovascular diseases (CVD) are the most common non-communicable disease (NCD). In 2019, CVD were responsible for an estimated 17.9 million deaths worldwide, of which more than three quarters occurred in low- and middle-income countries (LMIC) [1]. The increasing rate of deaths due to CVD remains a serious health concern. By 2030, 23 million people are expected to die from CVD, stroke and coronary heart diseases being the leading factors of these deaths. In sub-Saharan Africa (SSA), an increased prevalence rate of NCD, especially CVD, has been observed in the past 30 years. Ischemic heart diseases have now become a major leading cause of death in SSA, surpassing other major infectious diseases (malaria, tuberculosis and intestinal infections) [2].

Cardiometabolic diseases (CMD) are related to modifiable and non-modifiable risk factors (RF). Indeed, age, hypertension, diabetes, dyslipidemia, tobacco smoking, physical inactivity, high alcohol consumption, insufficient fruit/vegetable consumption and high fat intake are considered to be directly linked to the occurrence of CMD. High blood pressure (HBP) levels accelerate the risk of stroke and coronary heart disease. Likewise, people with diabetes are twice as likely to develop CVD. Physical inactivity is one of the lifestyle factors which promotes the onset of obesity, HBP, diabetes mellitus and an abnormal lipid profile. In SSA, HBP, type 2 diabetes, overweight and obesity were shown to be the most prevalent cardiometabolic risk factors (CMRF) [3].

Moreover, there has been a rapid increase of urbanization in SSA, as well as significant population growth and aging in the past decades. Rapid and unplanned urbanization has been shown to contribute to the increase of CMD prevalence due to the increase of RF [4]. As an example, unplanned urbanization is always characterized by the absence of spaces dedicated to physical activity. Furthermore, the urban environment provides an abundant market for the consumption of tobacco, alcohol and unhealthy food [5,6].

Previous studies performed in high- and low-income countries highlighted disparities in the prevalence and types of CMRF in urban versus rural settings. For instance, in Cameroon, a higher prevalence of obesity (17.1%) and hyperglycemia (10.4%) has been reported in urban areas compared to rural ones [7]. In a study carried out in Burkina Faso, HBP was found in 24.8% and 15.4% of individuals living in urban and rural areas, respectively [8]. Furthermore, differences in the rates of RF according to gender have been described as well. Obesity and abdominal obesity are more often observed in women while a higher tobacco consumption was found among men [9,10].

In Gabon, data on the burden of CMD and CMRF are scarce. Nevertheless, previous studies reported a non-negligible incidence of CVD (13.3%) and a relatively high prevalence of diabetes (19.6%) [11,12]. Furthermore, according to the WHO global health statistics, one in five people is considered obese and in 2018, 17% of deaths were related to CVD [13]. In the same way, a high prevalence of HBP was described in young students [14]. It was recently shown that in SSA, the increased morbidity and mortality rates due to CMD are a consequence of inefficient prevention programs and healthcare strategies [15]. The lack of data on CMD and their risk factors are key impediments to implement efficient control efforts. Recent data are required to develop awareness programs, to prioritize preventive measures and to adapt healthcare strategies according to urban and rural areas. As such, in order to better contextualize updated prevention and healthcare strategies, it is important to characterize the burden of CMRF according to sociodemographic factors (age, gender) and urbanization levels in Gabon.

Thus, the aim of this study was to estimate the prevalence rates of CMRF in urban and rural areas of Gabon.

## Material and methods

### Study sites

A cross-sectional study was carried out from September 2020 to November 2021 in two urban areas (Melen and Libreville in the Western Coast) and two rural areas (Bitam in the North and Koulamoutou in the Southeast) in Gabon. Melen is located in the fifth district of Libreville, the capital city. This district has 148,129 inhabitants. Koulamoutou is located 586.9 km from Libreville, in the Ogooué-Lolo province. Bitam is located in the north of Gabon, 592 km from Libreville, in the Woleu-Ntem province. The Ogooué-Lolo and Woleu-Ntem provinces are the fourth and seventh most populated provinces in Gabon with 65,771 and 154,986 inhabitants, respectively. In these rural areas, the main activities of the population are agriculture, fishing and hunting.

The study was performed in Melen at the Centre de Recherche Biomédicale en Pathogènes Infectieux et Pathologies Associées (CREIPA), in Libreville at the Centre Hospitalier Universitaire de Libreville (CHUL), in Koulamoutou at the Centre Hospitalier Régional Paul Moukambi (CHRPM), and in Bitam at the Centre Médical de Bifolossi.

### Study population

The study population was recruited through awareness campaigns in healthcare centers and in the community. Volunteers aged 18 years old and over, with negative malaria, HIV, hepatitis, tuberculosis tests, without any known underlying other chronic or infectious, nor inflammatory disease, who had been residing in each of the four study sites for at least two years, and who gave their consent to participate were included. Pregnant women were not included.

### Sampling and sample size calculation

A consecutive sampling approach was used to include healthy volunteers. Briefly, prior to the interview with the investigators and any testing, the participants were informed on the study objectives, procedures, risk and benefits, and that hepatitis, malaria and HIV testing will be performed prior to the inclusion. All volunteers who accepted to be tested, signed an informed consent form before any procedures (interview and biological tests).

The sample size was calculated with the formula used to estimate a sample proportion:

$${(\text{n = z}}^{\text{2}}{\text{p(1-p)/e}}^{2})$$

The reported prevalence of cardiovascular disease in Gabon, which was estimated to be 17%, was used in the absence of previous data on CMDRF [12]. The significance level was 5%. Considering a non-response rate of 10%, the minimum sample size was 239 participants from rural and urban areas, respectively.

### Data collection and physical examination

Data were collected using an adapted version of the standardized WHO stepwise survey questionnaire by a team composed of experienced medical doctors, nurses and biologists [16]. The questionnaire was developed and electronically administered using the Research Electronic Data Capture software (RedCap). The first phase of the study included a face to face interview with participants, during which they were asked about their socio-demographic data and their lifestyle characteristics such as tobacco and alcohol consumption, dietary habits (fruit, vegetable and salt consumption), physical activity. In addition, information on their past or current medication for HBP or diabetes was also recorded. In the second phase of the study, anthropometric measurements such as height, weight and waist circumference were taken. Height and weight were measured using a height gauge and an electronic scale, respectively. Participants were weighed without shoes and wearing only light clothing. Measurements were recorded to the nearest 0.5 centimeter for height and 0.1 kg for weight. Body mass index (BMI) was calculated according to the following formula: weight (kg)/ height squared (m^2^). Waist circumference was measured (in cm) in a standing position, by measuring the midpoint between the lower margin of the last palpable rib and the top of the iliac crest, according to the WHO recommendations [16]. Blood pressure (BP) and heart rate were measured in the sitting position, on the left arm of each participant using an automatic blood pressure monitor (OMRON M3 Comfort, Kyoto, Japon) after 3 minutes of rest. Three measurements were performed 3 minutes apart and the average values of the two closest values of BP and heart beats were recorded.

### Biological analysis

After having filled out the questionnaire form and performing the physical examination, blood samples were collected in the morning from participants. The day before blood sampling, participants were advised to have their last meal before 8 p.m. Blood samples were collected from those with overnight fasting of at least 12 hours, thus between 7 am and 10 am, to be able to include volunteers who had dinner between 7 pm and 10 pm the last night. Fasting blood glucose, total cholesterol (Total-C), HDL cholesterol (HDL-C), then LDL cholesterol (LDL-C) and triglyceride (TG) concentrations were measured using the PentraC200 analyzer (HORIBA ABX SAS, France). All the biological data were also recorded in RedCap. All participants who provided blood samples were also tested for malaria, HIV, and hepatitis. History of previous tuberculosis or exposure to tuberculosis were also checked. Those volunteers who were tested positive for HIV, hepatitis B and malaria or who were found exposed to tuberculosis, were excluded.

### Quality procedure

Throughout the study, measurements were performed by two independent technicians. Participants with HBP and high fasting glucose levels, detected during screening, were seen 15 days later by the study team. If the same trends were observed, they were classified as having HBP or hyperglycemia. In case of diagnosis confirmation, they were referred to the physician of the nearest health center or case management. Double data entry was carried out to minimize the risk of data entry errors.

### Definition of variables

The variables were defined based on WHO criteria [16]. Smokers were distributed into three groups: the first group included all tobacco (imported cigarettes, chewing tobacco, pipe, rolled cigarette) users; the second group consisted of daily smokers (i.e. individuals who smoked at least one cigarette per day); the third group consisted of occasional smokers (i.e. people who smoke between 1 to 3 cigarettes per week). Passive smokers were people who had experienced passive smoking at home or at work the 30 days prior to the day of the screening. Two groups were created for alcohol consumption: people who had consumed at least one alcoholic drink during the last 12 months, and those with excessive alcohol consumption, i.e. with more than 4 alcoholic drinks (12 g of ethanol) consumed per week. Based on different kinds of bottles or cans presented to the volunteers, the team estimated the amount of drinking. After each volunteer reported the number of bottles or cans he consumed, the physician estimated the standard quantity of drink per week and per day according to the number of glasses, cans or bottles of beer (650mL or 33cl/4.5°), wine (750mL or 10cL/12-14°), conventional glass (3-5cL) of liquor such as whisky (40°), pastis (45°), local drinks (1L or 25cL) like palm wine (15°), sugarcane wine (20–40°). The number of fruits and/or vegetables intake per day was also assessed: an intake of less than five fruits or vegetables per day was considered as insufficient. The survey team had a list of fruit and vegetables commonly consumed in the different areas. Apart from imported fruits and vegetables that are more often available in urban areas, a variety of local fruits and vegetables are consumed both in urban areas and in villages, depending on the seasons (oranges, mandarins, grapefruits, papayas, mangoes, guavas, passion fruits, sugar cane, watermelons, pineapples, avocados, safu, tomatoes, spinach, manioc leaves, tarot leaves, other green vegetables, asparagus, leeks, peppers…). The presence of a native speaker from each study area and of pictures of imported fruits and vegetables facilitated exchanges in the local language for the illiterate individuals. Salt consumption was also evaluated and individuals were ranked according to their habits: occasional or excessive salting when cooking, adding salt before eating or usual consumption of salty additives in food.

The physical activity was estimated using the Global Physical Activity Questionnaire (GPAQ) of WHO. Volunteers ‘occupation, household activities, walking and physical activities during their free time were assessed. Participant response interpretation was performed and presented in number Metabolic equivalent (MET) per week, which is the ratio of a person’s working metabolic rate relative to the resting metabolic rate per week. Insufficient physical activity was considered as less than 600 MET-minutes per week [17]. The sedentary lifestyle was characterized by the fact of spending 12 hours sitting or lying down over the course of a day.

A metabolic syndrome was diagnosed according to the definition established by the International Diabetes Federation in 2005 [18]. The diagnosis was based on the combination of at least three of the five following RF: elevated TG (>1.7 mmol/L); abdominal obesity (waist circumference ≥94 cm in men; ≥80 cm in women); systolic blood pressure ≥130 mmHg and/or diastolic blood pressure ≥85 mmHg or treated HBP, fasting blood glucose ≥5.56 mmol/L or treated diabetes mellitus; HDL-C <1.03 mmol/L in men, HDL-C <1.29 mmol/L in women). Abnormal lipid levels were classified according to the Adult Treatment Panel III criteria as follows: high total cholesterol (Total-C ≥ 6.2 mmol/L), low HDL cholesterol (HDL-C <1 mmol/L); high LDL cholesterol (LDL-C ≥4.1 mmol/L) and high triglyceride levels (TG >2.26 mmol/L) [19].

HBP was defined as systolic blood pressure ≥130 mmHg and diastolic blood pressure ≥80 mmHg or when participants followed a treatment for HBP. Pre-hypertension was defined as systolic blood pressure between 120–129 mmHg and diastolic blood pressure between 70–79 mmHg [14]. An elevated heart rate amounted to >80 beat/min. High fasting blood glucose was defined by venous blood glucose levels higher than 5.6 mmol/L after at least 12-hour fasting. Individuals on antidiabetic medication were considered has having diabetes mellitus. A BMI between 25 kg/m^2^ and 29 kg/m^2^ was considered as overweight, and obesity was defined as a BMI over 30 kg/m^2^, in line with the WHO guidelines [20]. Abdominal obesity was defined for a waist circumference ≥94 cm in men and ≥80 cm in women.

The Framingham risk score for coronary heart disease was used to estimate the 10-year cardiovascular risk of the volunteers. The factors used in the calculation included age, gender, diabetes, smoking, SBP, DBP, Total-C and HDL-C. The cardiovascular risk score was categorized as low (<10%), intermediate (10%-19%), and high (≥ 20%) as recommended [21].

The following eleven biological and behavioral RF were used to assess the number of CMRF in the different age groups of the study population: HBP, hyperglycemia, diabetes, obesity, overweight, abdominal obesity, dyslipidemia, tobacco use, excessive alcohol consumption, low fruit/vegetable consumption, insufficient physical activity. Metabolic syndrome was considered as an association of different RF.

Based on volunteer’s medical history, those who did not reported any underlying chronic disease, any specific medical condition, any underlying treatment medication other than antihypertensive or antidiabetic treatment during the last three months and who were not tested positive for malaria, HIV, hepatitis, tuberculosis tests, were considered apparently healthy and included in the study.

### Ethical considerations

Ethical clearance and approval were obtained from the National Ethics Committee under the reference PROT N°002/2020/PR/SG/CNE. The study also received administrative authorization from the Ministry of Health (which is the national regulatory authority), as well as from the local administrators of each the study areas. All individuals approached, including the authorities, were duly informed about the study’s objectives, procedures, expectations from potential participants, as well as the potential risks, the benefits and their rights. For the illiterate participants, a local community member, previously trained by the research team, gave them information in the local language. Written and signed informed consent was obtained from each participant. A fingerprint was used as a signature for the illiterates who were accompanied by a witness who also signed the consent form. According to the National Ethics Committee requirement, the participants, aged below 21 years old, who agreed to participate, signed a written assent form and their parents or legal guardians signed an informed consent form, after verification of their parental relationship. Participants with abnormal clinical or biological data were first examined by the study physician, afterwards, conducted with a medical report, to the site health facility for appropriate investigation and management in each site.

### Statistical analysis

Data were analyzed using Statistical Package for the Social Sciences (SPSS version 20) and Epi info (version 6). The data were further categorized according to each area, age group and gender. The normality of the data was checked by the Shapiro’s and Kolmogorov-Smirnov test for quantitative variables. Since the variables did not follow a normal distribution, medians were compared between groups using the Mann-Whitney U test and proportions were compared using a chi-squared test. Univariate logistic regression was performed to assess the unadjusted association between the studied variable and the presence of CMRF. Outcome variables were the CMD risk factors. Independent variables were areas of residence (urban, rural), gender (men, women) and age (18–35 years, 36–53 years and older than 53 years). All variables that were associated with the presence of at least one CMRF with a p<0.2 in the univariate analysis were included in the respective multivariable analysis. Associations of residence area, age and gender categories with the presence of at least one CMRF were assessed using different multinomial regression models. In the first model, Age and gender were considered as confound factors. When groups according to areas of residence and gender were predefined, the models only considered age as confound factors. Adjusted odds ratio (aOR) and cOR were reported at a 95% confidence interval (CI). All p-values are two-tailed, and p≤0.05 was considered statistically significant.

## Results

### Sociodemographic characteristics of the study population

**Fig 1:**
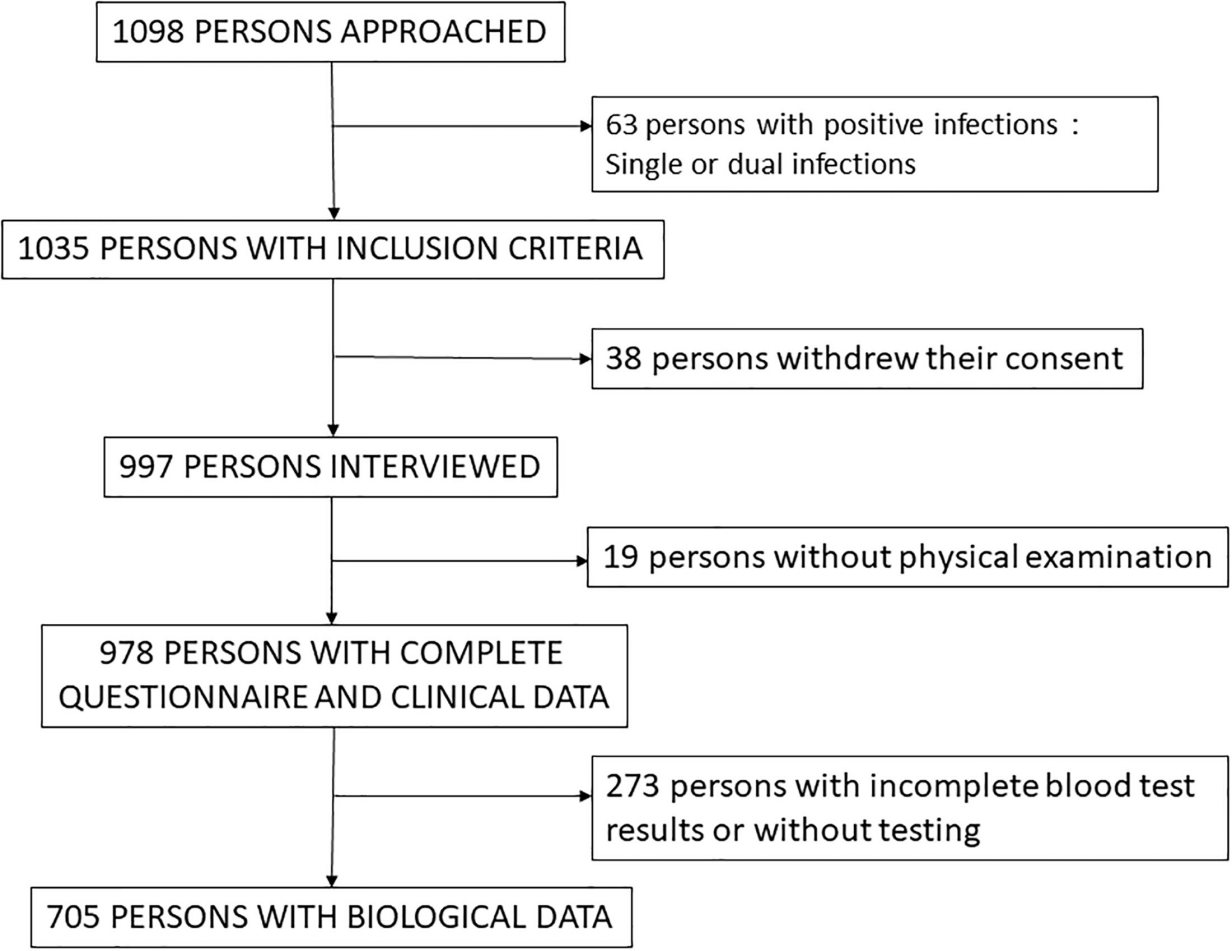
Study flow chart.

Overall, 1098 volunteers were approached, 63 had exclusion criteria (36 HIV positive, 15 HbS Ag positive, 42 malaria, some with a single positive test, some with at least two positive tests). Finally, 978 were included, 499 in urban areas and 479 in rural areas (Fig 1 and Table 1). The results of the lipid measurements were available for 705 participants, 458 volunteers (154 men; 304 women) in urban areas and 247 volunteers (100 men; 147 women) in rural areas.

**Table 1:** General characteristics of the study population.

Characteristics	Total (N = 978)	Urban area (n = 499)	Rural area (n = 479)
**Gender n(%)**			
**Men**	405 (41.4)	172 (34.5)	233 (48.6)
**Women**	573 (58.6)	327 (65.5)	246 (51.4)
**Age groups (years)**			
**18–35**	422 (43.1)	255 (51.1)	167 (34.9)
**36–53**	395 (40.4)	165 (33.1)	230 (48.0)
**>53**	161 (16.5)	79 (15.8)	82 (17.1)
**Mean age (±SD)**	39.5 ± 12.6	37.9 ± 13.4	41.0 ± 11.6
**Marital status n(%)**			
**Unmarried**	379 (38.8)	253 (50.7)	126 (26.3)
**Currently married**	261 (26.7)	134 (26.9)	127 (26.5)
**Living with a partner**	283 (28.9)	91 (18.2)	192 (40.1)
**Separated/Divorced/Widowed**	55 (5.6)	21 (4.2)	34 (7.1)
**Education level n(%)**			
**No formal education**	53 (5.4)	23 (4.6)	30 (6.3)
**Primary school**	45 (4.6)	22 (4.4)	23 (4.8)
**Middle school**	507 (51.9)	157 (31.5)	350 (73.0)
**High school**	147 (15.0)	93 (18.6)	54 (11.3)
**University education**	223 (22.8)	202 (40.5)	21 (4.4)
**Refused to answer**	3 (0.3)	2 (0.4)	01 (0.2)
**Occupation n(%)**			
**Public sector worker**	117 (12.0)	92 (18.4)	25 (5.2)
**Private sector employee**	137 (14.0)	30 (6.0)	107 (22.3)
**Self-employed**	282 (28.8)	70 (14.0)	212 (44.3)
**Worker/domestic worker**	72 (7.3)	55 (11.0)	17 (3.5)
**Students**	166 (17.0)	144 (28.9)	22 (4.6)
**Unemployed**	167 (17.1)	80 (16.0)	87 (18.2)
**Retired**	35 (3.6)	35 (3.6)	09 (1.9)
**Refused to answer**	2 (0.2)	2 (0.2)	0 (0.0)

Women represented almost 60.0% of the study population. The majority of the participants (83.5%) was aged less than 54 years old and the median age was 38 [28–50] years. More than half (55.6%) was in a relationship (married or living with a partner) and almost two thirds (66.9%) of the participants had at least a secondary school level education level (Table 1). Volunteers from urban areas were younger (37±13.4 vs 41± 11.6 years), more likely to be unmarried (50.7% vs 26.3%), and had a higher education level (40.5% vs 4.4%) than rural participants (*p* < 0.01) (Table 1).

### Prevalence of behavioral characteristics associated with cardiometabolic disease risk factors according to gender and area of residence

Overall, 156 (16.0%) participants smoked (Table 2). Men were more likely to be smokers than women, both in rural (44.2%vs 8.9%, p < 0.01), and urban (11.6% vs 3.4%, p < 0.01) settings (S1 Table). Participants from rural areas were more frequently tobacco users (Table 2). Indeed, daily users (29.4% vs 19.8%, *p* < 0.01) were more frequent in rural areas than in urban areas. Almost one quarter (24.7%) of the study population was exposed to passive smoking at work or at home (Table 2).

**Table 2:** Relationship between the presence of behavioral CMRF and area of residence.

Risk factors	Total	Urban	Rural	*p-value*
**TOBACCO CONSOMPTION n(%)**				
**All tobacco users**	156 (16.0)	31 (6.2)	125 (26.1)	**< 0.001**
**Daily smokers during previous 12 months**	106 (10.1)	14 (2.8)	92 (19.2)	**< 0.001**
**Occasional Tobacco users**	50 (5.1)	17 (3.4)	33 (6.9)	**0.013**
**Passive tobacco users during previous 30 days**	240 (24.5)	99 (19.8)	141 (29.4)	**< 0.001**
**ALCOHOL CONSUMPTION**				
**Previous 12 months**	640 (65.4)	329 (65.9)	311 (64.9)	0.741
**Excessive consumption during past 7 days**	141 (14.4)	48 (9.6)	93 (19.4)	**< 0.001**
**FRUITS AND VEGETABLES INTAKE**				
**Number of fruits/days(m±SD)**	1.27 ± 1.2	1.27 ± 1.3	1.27 ± 1.1	0.197
**Number of vegetables/day**	1.43 ± 0.8	1.36 ± 0.8	1.50 ± 0.8	0.235
**Insufficient fruits/vegetables intake n(%)**	859 (87.8)	432 (86.6)	427 (89.1)	0.218
**SALT CONSUMPTION n(%)**				
**Always/often adds additional salt during meals**	164 (16.8)	91 (18.2)	73 (15.2)	0.209
**Usual consumption of salty additives in food**	320 (32.7)	121 (24.2)	199 (41.5)	**< 0.001**
**PHYSICAL ACTIVITY n(%)**				
**Low**	225 (23.0)	147 (29.5)	78 (16.3)	**< 0.001**
**Sedentary lifestyle**	88 (9.0)	45 (9.0)	43 (9.0)	0.982

Alcohol consumption was found to be more prevalent than smoking among the general population, with men being the most frequent consumers in all the study areas (S1 Table). Less than 15% of the study participants had excessive consumption. Rural inhabitants (19.4% vs 9.6% in urban areas, *p* < 0.01) and men (30% vs 9.4% in women, *p* < 0.01) were more often excessive alcohol consumers (Tables 2 and S1).

Rural residents had higher rate of food with added salty additives intake compared to urban residents (41.5% vs 24.2%; p < 0.01) (Table 2). This trend was also observed among women residing in urban areas (S1 Table). The mean number of fruit and vegetables consumed was 2.8 ± 2.3 /day. Only 12.2% (n = 119/978) of volunteers ate the recommended five or more fruits and/or vegetables per day (Table 2). Neither age nor location statistically influenced the quantity of fruit and vegetable intake. However, women residing in urban areas more often ate fruits and vegetables compared to men, as indicated in S1 Table. The rate of insufficient physical activity was the highest in urban areas (29.5% vs 16.3%; *p* < 0.01), and in women (26.0%, n = 149/573 vs 18.8%, n = 76/405; *p* <0.01) (Tables 2 and S1).

In multivariate analysis, men were more likely to be tobacco users in both urban (AOR: 4.0[1.8–8.4], *p* < 0.01) and rural areas, (AOR: 8.0[4.9–13.5], *p* < 0.01), respectively (S2 Table). Rural participants were two fold (AOR: 1.96; 95%CI [1.33–2.88], *p* < 0.01), and men four fold (AOR: 4.1; 95%CI [2.49–6.95], *p* < 0.01) at a higher risk of being excessive alcohol consumers (Tables 3 and S2).

**Table 3:** Multivariate analysis of behavioral risk factors according to study area.

Variables	Areas	Crude 0R (95%CI)	Adjusted 0R (95%CI)	*p-value*
**Tobacco smoking**				
	Urban	Ref		
	Rural	5.33 (3.51–8.08)	4.78 (3.1–7.3)	**< 0.001**
**Daily tobacco smoking**				
	Urban	Ref		
	Rural	8.23 (4.62–14.67)	7.97 (4.38–14.53)	**< 0.001**
**Occasional tobacco smoking**				
	Urban	Ref		
	Rural	2.09 (1.15–3.81)	2.43 (1.32–4.47)	**0.004**
**Passive tobacco smoking**				
	Urban	Ref		
	Rural	1.68 (1.25–2.26)	1.7 (1.27–2.31)	**< 0.001**
**Excessive alcohol consumption**				
	Urban	Ref		
	Rural	2.26 (1.55–3.29)	1.96 (1.33–2.88)	**0.0007**
**Low physical activity**				
	Urban	2.14 (1.57–2.92)	2.1 (1.54–2.93)	**< 0.001**
	Rural	Ref		
**Usual consumption of salty additives in food**				
	Urban	Ref		
	Rural	2.22 (1.68–2.91)	2.39 (1.8–3.1)	**< 0.001**

Ref: Reference; AOR adjusted for study area.

### Prevalence of anthropometrics and biological cardiometabolic disease risk factors according to gender and area of residence

HBP, diabetes, abdominal obesity, obesity, high LDL-C level, high Total-C level and metabolic syndrome were significantly more common in urban than in rural areas (Table 4).

**Table 4:** Relationship between anthropometric, biological, and number of CMRFs with area of residence.

Risk factors	Total	Urban	Rural	p-value
**HBP n(%)**	486 (49.7)	297 (59.5)	189 (39.5)	**< 0.001**
**Pre-hypertension**	99 (10.1)	30 (6.0)	69 (14.4)	**< 0.001**
**Elevated heart rate**	345 (35.3)	125 (25.1)	220 (45.9)	**< 0.001**
**Diabetes**	53 (5.4)	51 (10.2)	02 (0.4)	**< 0.001**
**Abdominal obesity**	288 (29.4)	171 (34.3)	117 (24.4)	**< 0.001**
**Overweight**	256 (26.2)	132 (26.5)	124 (25.9)	0.840
**Obesity**	233 (23.8)	142 (28.5)	91 (19.0)	**< 0.001**
**Low HDL-C level**	163 (23.1)	58 (12.7)	105 (42.5)	**< 0.001**
**High Triglycerides level**	14 (2.0)	09 (2.0)	5 (2.0)	0.818
**High LDL-C level**	45 (6.1)	44 (9.6)	01 (0.4)	**< 0.001**
**High Total-C**	45 (6.4)	45 (9.8)	00 (0.0)	**—**
**Hyperglycemia**	197 (20.1)	113 (22.6)	84 (17.5)	0.464
**Metabolic Syndrome**	122 (12.5)	83 (16.6)	39 (8.1)	**< 0.001**
**NUMBER OF FACTORS**				
**None**	06 (0.6)	03 (0.6)	03 (0.6)	**0.719**
**1**	97 (9.9)	53 (10.6)	44 (9.2)	0.452
**2**	198 (20.2)	202 (40.5)	96 (20.0)	**< 0.001**
**3**	275 (28.1)	120 (24.0)	155 (32.4)	**0.003**
**4**	253 (25.9)	127 (25.5)	126 (26.3)	0.760
**5**	112 (11.5)	69 (13.8)	43 (9.0)	**0.017**
**More than 5**	37 (3.8)	25 (5.0)	12 (2.5)	**0.040**

HBP: High blood pressure; LDL-C: Low density lipoprotein-cholesterol; HDL-C: High density lipoprotein-cholesterol; Total-C: Total cholesterol.

When considering participants under medication for hypertension and pre-hypertension, the global prevalence of HBP was 59.8% according to Table 4. Considering only the 486 respondents who had a HBP, 97 (20.0%) were on medication, thus 39.7% (n = 386/978) of the whole study participants were either untreated or undiagnosed. Men were more likely to have HBP (43.3% vs 35.8%, *p* = 0.01) or pre-hypertension (12.8% vs 2.4%, *p* < 0.01) than women (S3 Table). However, while rural inhabitants had a lower prevalence of HBP (39.5% vs 59.5%, *p* < 0.01), they had more often pre-hypertension (14.4% vs 6.0% in urban inhabitants, *p* < 0.01) (Table 4). The same trends was observed between volunteers aged less than 36 years old and older participants (10.6% vs 3.9% respectively; *p* < 0.01). However, 36.5% of participants aged less than 36 years had HBP, compared to 62.2% of participants aged between 36 and 53 years old and 79.9% of participants who were older than 53 years old. Obesity or overweight was more prevalent among workers (54.2%) (public sector worker, private sector employee, self-employed and worker/domestic worker) than students (30.7%) and unemployed (52.7%), (*p* < 0.01).

The mean waist circumference and the mean BMI were 84.82 ± 15.53 cm and 26.50 ± 6.64 kg/m^2^ respectively, in the overall study population. Abdominal obesity was found in more than 20% of the participants, while the overall prevalence of overweight and obesity was 50.0% (Table 4). Living in urban areas (28.5% vs 19.0% in rural settings, *p* < 0.01), and being a woman (34.6% vs 16.9% in men, *p <* 0.001), were associated with obesity (Tables 4 and S3). According to age, a higher prevalence of obesity was seen in adults aged 36–53 years old (34.5%) compared to the oldest (29.8%) and the youngest (16.4%; *p* < 0.01). The prevalence rate of overweight was comparable between the three age groups, ranging from 25.0% to 29.1%.

The multivariate analysis confirmed that inhabitants from rural areas were 1.5 times more at risk to have pre-hypertension (AOR: 1.5; 95%CI [0.88–2.63]) (Table 5). Living in urban areas (AOR: 1.89; 95%CI [1.31–2.63], p < 0.01) and woman (AOR: 4.2; 95%CI [3.54–7.14], p < 0.01) were risk factors for obesity (Tables 5 and S4).

**Table 5:** Multivariate analysis of biological CMRFs according to study area.

Variables	Areas	Crude 0R (95%CI)	Adjusted 0R (95%CI)	P-value
**HBP**				
	Urban	2.25 (1.74–2.91)	3.0 (2.23–4.12)	**< 0.001**
	Rural	Ref		
**Pre-hypertension**				
	Urban	0.4 (0.24–0.59)	0.6 (0.41–1.08)	**0.105**
	Rural	Ref		
**Diabetes**				
	Urban	27.15 (6.57–112.18)	52.2 (12.3–221.3)	**< 0.001**
	Rural	Ref		
**Metabolic syndrome**				
	Urban	2.25 (1.52–3.36)	2.46 (1.61–3.76)	**< 0.001**
	Rural	Ref		
**Obesity**				
	Urban	1.69 (1.25–2.28)	1.86 (1.31–2.63)	**< 0.001**
	Rural	Ref		
**Abdominal obesity**				
	Urban	1.61 (1.22–2.13)	1.55 (1.13–2.13)	**0.006**
	Rural	Ref		
**Low HDL—c level**				
	Urban	0.5 (0.33–0.66)	0.2 (0.13–0.29)	**< 0.001**
	Rural	Ref		
**High LDL-c level**				
	Urban	46.2 (6.34–336.8)	35.0 (4.7–257.4)	**< 0.001**
	Rural	Ref		

HPB: High blood pressure; LDL-C: Low density lipoprotein-cholesterol; HDL-C: High density lipoprotein-cholesterol; Ref: Reference; AOR adjusted for area.

The mean (±SD) levels of Total-C, LDL-c, HDL-C, TG were 4.89 (±1.29) mmol/L, 2.43 (±1.07) mmol/L, 1.36 (± 0.52) mmol/L and 0.82 (± 0.52) mmol/L, respectively. Low HDL-C level (23.1%) was the most frequent dyslipidemia, while the rates of high levels of the other lipid parameters were below 7.0% (Table 4). The highest rate of low HDL-C was found in rural areas. High LDL-C and hypercholesterolemia were more frequent in inhabitants from urban sites. However, no difference was observed between men and women for high Total-C levels (5.5% in men vs 6.3% in women; *p* = 0.47), and for high LDL-C (6.7% in men vs 6.2% in women; *p* = 0.92) (S3 Table).

Our results show a higher trend of hyperglycemia (3.2% vs 1.3%; *p* = 0.10) and low HDL-C (26.8% vs 21.1%; *p* = 0.09) in men compared to women. The mean Total-C value was 6.9 (± 0.7) mmol/L in the group of volunteers with a high Total-C level, the mean HDL-C rate was 0.69 (± 0.23) mmol/L in participants with low HDL-C level, the mean LDL-C level was 4.91 (± 0.93) mmol/L in participants with high LDL-C, and finally, the mean TG was 3.26 (± 0.49) mmol/L in the group of volunteers with hypertriglyceridemia.

The overall prevalence of diabetes was 5.4% and the prevalence of high fasting blood glucose levels was also higher (Table 4). Hyperglycemia rate was comparable in urban and rural areas, while 51 out of the 53 volunteers under diabetes medication lived in urban sites (*p* < 0.01). The frequency of both abnormalities was not statistically different according to gender. The overall prevalence of metabolic syndrome was higher in women (21.7%) than in men (10.0%) (*p* < 0.01) (S3 Table).

### Framingham cardiovascular risk score according to urban and rural areas

The Framingham risk score for coronary heart disease was assessed only in the group of the 505 participants aged over 30 years old (296 in urban areas and 209 in rural areas). Overall, 20 (4.0%) participants had a high score, and among these 18 (6.1%) lived in urban settings while only 2 (1.0) resided in rural areas (*p* < 0.01). The rates of people with a moderate score were comparable in the two areas (12.5% in urban area vs 11.0% in rural area, *p* = 0,61). The prevalence of a high risk score was significantly lower in men (2.5% vs 6.4% in women; *p* = 0.03), and in older participants (13.7% vs 1.4% in those aged between 36–53 years old; *p <* 0.01).

### Number of cardiometabolic disease risk factors according to gender, age group and area of residence

Overall, 972 (99.4%) participants had at least one modifiable CMD risk factor. Participants frequently had three or four RF (54.0%) and those with the highest number of risk factors resided in urban areas (Table 4).

**Fig 2:**
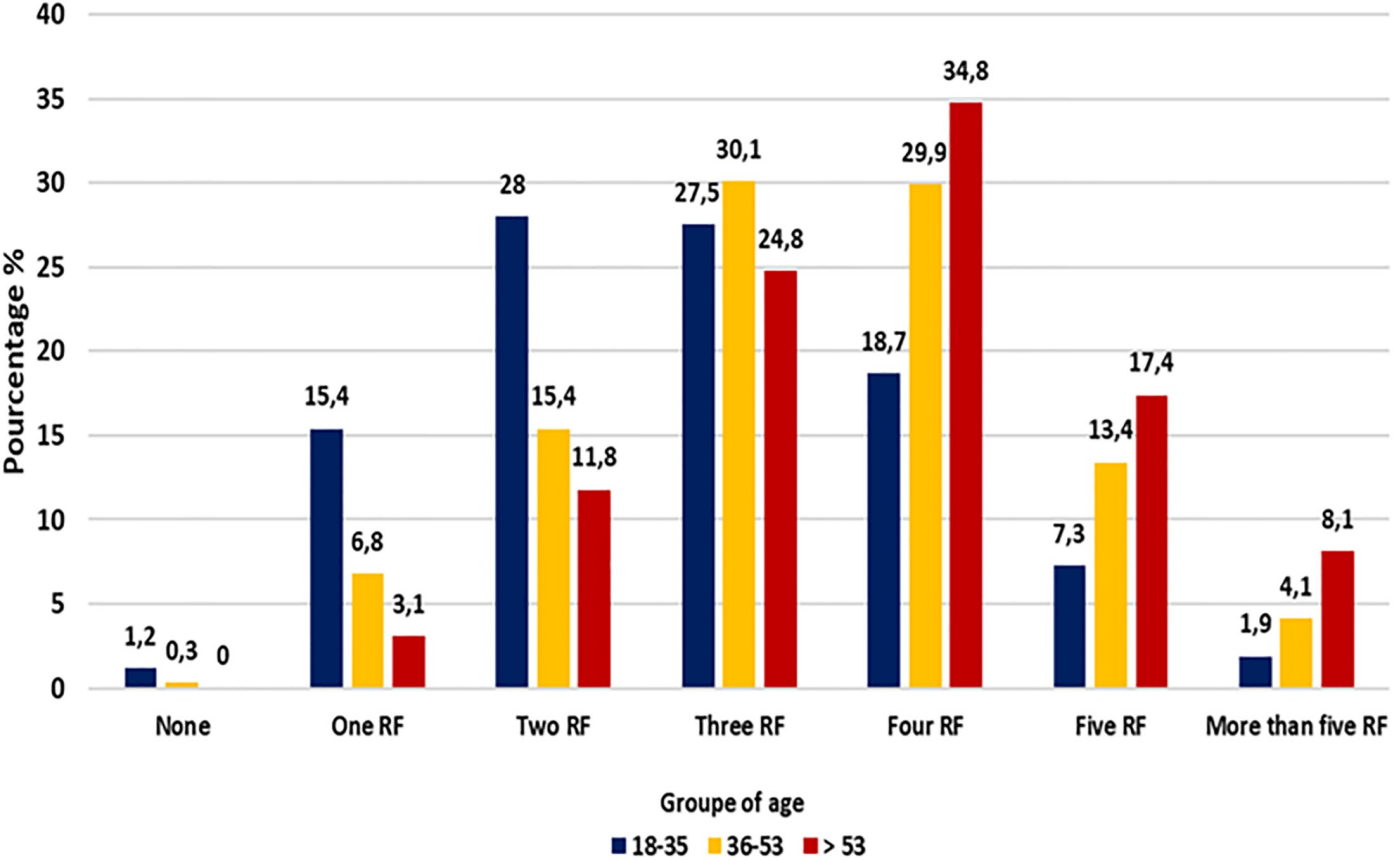
Number of cardiometabolic disease risk factors according to age groups.

The number of CMRF per individual significantly varied according to age (*p* < 0.01) (Fig 2). The majority of healthy volunteers aged under 36 years old (70.9%; n = 299/422) had three RF or less; two thirds of the oldest participants (60.3%, n = 97/161) had four RF or more, while the middle-aged volunteers had more frequently (60.0%, n = 237/395) three to four CMRF (Fig 2).

## Discussion

This study shows a considerable high prevalence of NCD risk factors in people living in urban (Libreville and Melen located in the West) and rural (Bitam in the North and Koulamoutou in the South-East settings in Gabon.

The observed prevalence of daily tobacco use was higher than the rates recorded in young Gabonese students (19.2% vs 14%), in Kenyan adults (13.5%) and in global reports from SSA [14,22–24]. The fact that men are more likely to be smokers than women is commonly reported worldwide [9,25]. However, an unusual higher prevalence of daily tobacco users in rural areas (19.2%) compared to urban settings (2.8%) was observed. In SSA countries, the smoking prevalence rate was 40% in urban areas and 20% in rural areas [26]. Culturally, the consumption of chewing tobacco is very common in rural communities. Furthermore, the geographical location of the study area, Bitam and Koulamoutou, offers convenient access to more affordable tobacco products, including cigarettes, from neighboring countries; Cameroon and Equatorial Guinea. It seems that awareness campaigns are insufficient in the country and do not reach rural communities where school attendance is lower.

Excessive alcohol consumption was more prevalent in men and in rural settings as observed elsewhere [9,10]. Alcohol and tobacco consumption in women is traditionally considered as abnormal or incorrect (immoral) in Gabon, while it is more accepted for men. This could explain the difference highlighted according to gender. Alcohol consumption was more often reported by rural inhabitants. Each family has a plantation, allowing them to produce their own palm wine or sugar cane liqueur, whose alcohol level can exceed 30%. These two drinks, which are very popular in local communities, are very often consumed.

The prevalence of low fruit and vegetable intake is in line with reports from Tanzania (82%) and Ethiopia (95%) [26,27]. There was no difference according to gender or the geographic location. Most participants in this study (74%) did not have a sufficient and regular income which would allow them to buy and consume several fruits and vegetables on a daily basis. In addition, the average Gabonese meal consists of a starchy food and meat or fish for lunch, which is the main meal of the day.

The prevalence rate of high salt intake in cooking and dietary habits reached 42% in total when the consumption of processed food with salt was combined with the addition of salt during meals. The higher prevalence observed in rural areas can be attributed to the lack of preventive information regarding the risks of CVD associated with more than 5g of salt consumption per day. Additionally, the scarcity of cardiologists in rural areas, who typically offer education and counseling on this matter, further contributes to this situation.

The overall prevalence of insufficient physical activity is 22% in SSA, which is in line with the results in our present study (23%) [28]. The higher proportion of women and urban participants with insufficient activity seems to be common in LMIC [28]. The sedentary lifestyle (at home and at work) in urban cities, and the fact that men do more physical work compared to women, might partly be in cause.

The high prevalence rate of overweight and obesity within the study population is a matter of great concern, as it reached 50%. This is very high compared to reports from other SSA countries such as Zambia (24.4%) and Mozambique (30%) [29,30]. The predominance of insufficient physical activity and intake of salty food among women and the urban population justifies the observed higher predominance of overweight/obesity and central obesity in these two groups. Our results are in accordance with other data in African countries where the prevalence rate of overweight/obesity has doubled within the last two decades, with the highest burden found in women and urban inhabitants [31,32]. In Libreville, the work is carried out in a continuous day with a break of less than an hour. Workers, therefore, use to have lunch in fast food outlets, where fried foods and meals rich in oil and salt are most frequently available. This poor lifestyle may explain the higher prevalence of obesity/overweight in the group of patients aged 35 to 53 years old, which is the most professionally active in our study population.

Hypertension is the leading CMRF in Africa, its prevalence is the highest in the world, ranging from 17% to 70% [33]. Almost half (49.7%) of the interviewed persons in our study had a high blood pressure, which is consistent with a previous report from the main hospital in Libreville, but considerably higher compared to studies from Oman [34,35]. Although the differences could be due to the cross-sectional study design and other parameters such as the stress of the participants during the physical examination, these findings should alert the physicians, health workers, and policy makers. These findings also suggest that more than 25% of the population were not aware of their health status or were undiagnosed, and thus would remain untreated, increasing the risk of ischemic attack. The prevalence of HBP was lower in rural settings, however it reached 39% in these areas where it is known that awareness campaign and annual systematic screening and treatment are not often accessible. Moreover, the rate of prehypertension, which is also a risk factor for cardiometabolic diseases, was found to be higher at Bitam and Koulamoutou [36]. A recent study conducted in Libreville revealed an HBP prevalence rate of nearly 20% among adolescents [14]. All together, these data suggest that HBP is a major public health problem in Gabon.

The prevalence rate of diabetes was 5.4% in this study, which is in line with studies from Cameroon (5.6%), Burkina Faso (5.2%) [37,38]. Not surprisingly, 97% of the diabetic participants lived in urban areas and, as such, the prevalence rate was higher (10.2%) than in rural areas. The prevalence of hyperglycemia was much higher than that of diabetes, and it was comparable in urban and rural settings. Beyond the deaths directly attributable to diabetes, hyperglycemia is responsible for a heavy burden of mortality [39]. It is therefore urgent to implement a strategy to treat patients in the pre-diabetes stage in order to avoid a progression of the disease to diabetes and its medical and economic consequences.

The burden of dyslipidemia is increasing in SSA [22]. Low HDL-C level was the most frequent detected dyslipidemia (23.1%), which is consistent with data from SSA (20–40%) [40]. High Total-C (6.4%) and hypertriglyceridemia (6.3%) prevalence rates were lower than rates from other SSA countries [41,42]. However, the concerning prevalence rate of the metabolic syndrome (12.5%), which is a precursor of CVD and diabetes, is in line with rates from SSA (16%) [42]. Women and urban populations are the most concerned. This is not surprising because related behavioral risk factors were also found to predominate in these groups. Rates of pre-HBP (14.4% vs 6.0%), elevated heart rate (45.9% vs 25.1%) and low HDL-C (42.5% vs 12.7%) levels were predominant in rural areas. These results could reflect an ongoing epidemiologic transition, in rural areas.

More than 80% of the participants had at least two NCD risk factors, although people living in urban sites (18.8%) were more likely to have more than three RF than rural inhabitants (11.5%). In Kenya, the majority (75%) of adults screened had at least four RF [43]. As observed elsewhere, increasing age and urban residence were associated with a higher number of CMRF [29].

According to the Framingham risk score, people at a high risk of suffering from a cardiovascular event in next 10 years were more likely to live in urban areas than in rural areas (6.08% vs 0.95%), as reported in Myanmar [9]. These results are not surprising as the variables considered in the calculation of the Framingham risk score were found in high prevalence rates in urban sites.

Urban inhabitants had more risk factors and older people had a higher number of RF. It has been suggested that the risk of cardiovascular events increase with particular combinations of risk factors as well as their total number [44,45]. As an example, hypertension associated with the elderly may be combined with several others RF. Thus, strategies to reduce RF in the population should be adapted to target the population according to the areas.

This is the first study which assessed CMRF both in urban and rural areas of Gabon, where no data were previously recorded. A standardized approach was used, making the generated data comparable to other countries. Data was collected by well-trained team members, and the presence of field workers living in each of the study area and speaking the native language, facilitated exchanges with the populations in rural localities. The recorded data were self-reported through face-to face interviews on personal lifestyle. Thus, the participants may tend to say what is acceptable or correct instead of telling the truth. As a consequence, alcohol and tobacco consumption could be under-reported. Moreover, the measurement of salt in urine was not performed to assess the reliability of the volunteers estimates of their salting habits. Despite these limitations, this study provides useful baseline information on the burden of CMRF and could be used by policy makers and control programs.

## Conclusion

The prevalence of modifiable CMD risk factors was high, both in urban and rural study sites. Gender differences in risk factor prevalence was observed with the predominance of tobacco use and alcohol consumption in men, and of insufficient activity, obesity and metabolic syndrome in women, irrespective of the study area. Apart from physical activity, other behavioral NCD risk factors predominated in rural settings. The high prevalence of multiple RF per individual indicates the urgent need to implement effective preventive programs as well as the detection, treatment and prevention of related cardiovascular and metabolic diseases. Other surveys with larger sample populations and specific groups such as youth, elderly, pregnant women and HIV-infected persons should also be performed.

## Supporting information

S1 Table Relationship between the presence of behavioral CMRF and gender. p-value^a^:Men-Women comparison in urban areas; P-value^b^:Men-Women comparison in rural areas.
(DOCX)

S2 Table Multivariate analysis of behavioral risk factors according to gender. AOR adjusted for age; *P-value*^a^:Men-Women comparison in urban areas; *P-value*^*b*^:Men-Women comparison in rural areas.
(DOCX)

S3 Table Relationship between anthropometric, biological, and number of CMRF with gender. P-value^a^:Men-Women comparison in urban areas; P-value^b^:Men-Women comparison in rural areas; HBP: High blood pressure; LDL-C: Low density lipoprotein-cholesterol; HDL-C: High density lipoprotein-cholesterol; Total-C: Total cholesterol.
(DOCX)

S4 Table Multivariate analysis of biological CMRF according to gender. AOR adjusted for age; P-value^a^:Men-Women comparison in urban areas; P-value^b^:Men-Women comparison in rural areas; Ref: Reference; HBP: High blood pressure.
(DOCX)

## References

[1] World Health Organization. Cardiovascular diseases report 2021[Internet].[cited 15 Jun 2022]. https://www.who.int/news-room/fact-sheets/detail/cardiovascular-diseases-(cvds).

[2] Institute for Health Metrics and Evaluation. GBD Compare Data Visualization 2020 [Internet].[cited 12 Feb 2024]. http://vizhub.healthdata.org/gbd-compare.

[3] Mudie K, Jin MM, Kendall L, Addo J, dos-Santos-Silva I, Quint J, et al. Non-communicable diseases in sub-Saharan Africa: a scoping review of large cohort studies. J Glob Health. 2019 Dec; 9(2). doi: 10.7189/jogh.09.020409 . 31448113 PMC6684871

[4] Teo KK, Rafiq T. Cardiovascular Risk Factors and Prevention: A Perspective From Developing Countries. CJC. 2021 May; 37(5):733–43. doi: 10.1016/j.cjca.2021.02.009 . 33610690

[5] Popkin BM, Adair LS, Ng SW. Global nutrition transition and the pandemic of obesity in developing countries. Nutr Rev.2012 Jan; 70(1):3–21. doi: 10.1111/j.1753-4887.2011.00456.x . 22221213 PMC3257829

[6] Sliwa K, Acquah L, Gersh BJ, Mocumbi AO. Impact of socioeconomic status, ethnicity, and urbanization on risk factor profiles of cardiovascular disease in Africa. Circulation. 2016 Mar; 133(12):1199–208. doi: 10.1161/CIRCULATIONAHA.114.008730 . 27002082

[7] Sobngwi E, Mbanya JN, Unwin N, Kengne A, Fezeu L, Minkoulou E, et al. Physical activity and its relationship with obesity, hypertension and diabetes in urban and rural Cameroon. Int J Obes Relat Metab Disord. 2002 Jul; 26(7):1009–16. doi: 10.1038/sj.ijo.0802008 . 12080456

[8] Soubeiga JK, Millogo T, Bicaba BW, Doulougou B, Kouanda S. Prevalence and factors associated with hypertension in Burkina Faso: a countrywide cross-sectional study. BMC Public Health. 2017 Jan; 17(1):64. doi: 10.1186/s12889-016-3926-8 . 28077112 PMC5225558

[9] Htet AS, Bjertness MB, Sherpa LY, Kjøllesdal MK, Oo WM, Meyer HE, et al. Urban-rural differences in the prevalence of non-communicable diseases risk factors among 25–74 years old citizens in Yangon Region, Myanmar: a cross sectional study. BMC Public Health. 2016 Dec; 16(1):1–12. doi: 10.1186/s12889-016-3882-3 . 27919240 PMC5139102

[10] Houehanou YCN, Lacroix P, Mizehoun GC, Preux P-M, Marin B, Houinato DS. Magnitude of cardiovascular risk factors in rural and urban areas in Benin: findings from a nationwide steps survey. PLoS One. 2015 May;10(5):e0126441. doi: 10.1371/journal.pone.0126441 . 25945498 PMC4422555

[11] Ayo Bivigou E, Igala M, Manomba C, Allognon C, Ndoume F, Yekini C, et al. Profile of cardiovascular manifestations in COVID-19 patients at the Libreville university hospital center, Gabon. Arch Cardiovasc Dis Suppl. 2022 Jan; 14(1):127–127. doi: 10.1016/j.acvdsp.2021.10.006

[12] Ngoungou EB, Aboyans V, Kouna P, Makandja R, Nzengue JEE, Allogho CN, et al. Prevalence of cardiovascular disease in Gabon: a population study. Arch Cardiovasc Dis. 2012 Feb;105(2):77–83.. 22424325 10.1016/j.acvd.2011.12.005

[13] Organisation mondiale de la Santé. Bureau régional de l’Afrique. Stratégie de coopération OMS dans le pays 2016–2021: Gabon. World Health Organization Regional Office for Africa 2016 [Internet].[cited 12 Feb 2024] https://apps.who.int/iris/handle/10665/254892.

[14] Bivigou EA, Ditombi BM, Nguema OM, Moutongo R, Pongui B, Ekomi BB, et al. Hypertension and prehypertension: prevalence and associated factors in Gabonese Youth and Adolescents. 2020 [Internet].[cited 12 Feb 2024]. https://assets.researchsquare.com/files/rs-24889/v2/84500846-5261-445c-9b73-ce5361b5f540.pdf?c=1631845768.

[15] World Health Organization. Global status report on noncommunicable diseases 2014; one more landmark step in the combat against stroke and vascular disease.2014 [Internet].[cited 12 Feb 2024].https://apps.who.int/iris/handle/10665/148114. 10.1161/STROKEAHA.115.008097 25873596

[16] World Health Organization. The WHO STEPwise approach to chronic disease risk factor surveillance (STEPS). Geneva, Switzerland: WHO. Contract No.: Document Number. 2008[Internet].[cited 25 Mar 2020]. https://www.who.int/teams/noncommunicable-diseases/surveillance/systems-tools/steps/manuals.

[17] World Health Organization. Global physical activity questionnaire (GPAQ). analysis guide, 2012. https://www.who.int/ncds/surveillance/steps/GPAQ_EN.pdf [Accessed 17 Oct 2019].

[18] Zimmet P, Alberti K, Shaw J. International Diabetes Federation: the IDF consensus worldwide definition of the metabolic syndrome. Diabet Med. 2006 May; 50:31–3. 10.1111/j.1464-5491.2006.01858.x 16681555

[19] National Cholesterol Education Program (NCEP). Expert Panel on Detection, Evaluation, and Treatment of High Blood Cholesterol in Adults (Adult Treatment Panel III) final report. 2002 [Internet]. [12 Dec 2023] https://www.aefa.es/wp-content/uploads/2014/04/NECP-guidelines-.pdf. 12485966

[20] World Health Organization. 2021. Obesity and overweight. [Internet].[cited 18 July 2023]. https://www.who.int/news-room/fact-sheets/detail/obesity-and-overweight.

[21] Wilson PW, D’Agostino RB, Levy D, Belanger AM, Silbershatz H, & Kannel WB. Prediction of coronary heart disease using risk factor categories. Circulation.1998 May; 97(18). 1837–1847. doi: 10.1161/01.cir.97.18.1837 . 9603539

[22] World Health Organization. WHO global report on trends in prevalence of tobacco use 2000–2025. 2021[Internet].[cited 15 Dec 2022]. https://www.who.int/publications/i/item/who-global-report-on-trends-in-prevalence-of-tobacco-use-2000-2025-third-edition.

[23] Mwangi KJ, Mwenda V, Gathecha G, Beran D, Guessous I, Ombiro O, et al. Socio-economic and demographic determinants of non-communicable diseases in Kenya: a secondary analysis of the Kenya stepwise survey. Pan Afr Med J. 2020 Dec; 37. doi: 10.11604/pamj.2020.37.351.21167 . 33796165 PMC7992900

[24] Peltzer K, Pengpid S. Tobacco use, beliefs and risk awareness in university students from 24 low, middle and emerging economy countries. Asian Pac J Cancer Prev. 2014;15(22):10033–8. doi: 10.7314/apjcp.2014.15.22.10033 . 25520065

[25] Reitsma MB, Fullman N, Ng M, Salama JS, Abajobir A, Abate KH, et al. Smoking prevalence and attributable disease burden in 195 countries and territories, 1990–2015: a systematic analysis from the Global Burden of Disease Study 2015. Lancet. 2017 May; 389(10082):1885–906. doi: 10.1016/S0140-6736(17)30819-X . 28390697 PMC5439023

[26] Msambichaka B, Eze IC, Abdul R, Abdulla S, Klatser P, Tanner M, et al. Insufficient fruit and vegetable intake in a low-and middle-income setting: a population-based survey in semi-urban Tanzania. Nutrients. 2018 Feb; 10(2):222. doi: 10.3390/nu10020222 . 29462925 PMC5852798

[27] Motuma A, Demissie Regassa L, Gobena T, Teji Roba K, Berhane Y, Worku A. Almost all working adults have at least one risk factor for non-communicable diseases: Survey of working adults in Eastern Ethiopia. PloS one. 2022 Feb; 17(2):e0264698. doi: 10.1371/journal.pone.0264698 . 35226698 PMC8884490

[28] Pengpid S, Peltzer K, Kassean HK, Tsala Tsala JP, Sychareun V, Müller-Riemenschneider F. Physical inactivity and associated factors among university students in 23 low-, middle-and high-income countries. Int J Public Health. 2015 Jul; 60(5):539–49. doi: 10.1007/s00038-015-0680-0 . 25926342

[29] Pengpid S, Peltzer K. Prevalence and correlates of multiple non-communicable disease risk factors among adults in Zambia: Results of the first national STEPS survey in 2017. Pan Afr Med J. 2020 Nov; 37:265. doi: 10.11604/pamj.2020.37.265.25038 . 33598080 PMC7864270

[30] Fontes F, Damasceno A, Jessen N, Prista A, Silva-Matos C, Padrão P, et al. Prevalence of overweight and obesity in Mozambique in 2005 and 2015. Public Health Nutr. 2019 Dec; 22(17):3118–26. doi: 10.1017/S1368980019002325 . 31453793 PMC10260445

[31] Amugsi DA, Dimbuene ZT, Mberu B, Muthuri S, Ezeh AC. Prevalence and time trends in overweight and obesity among urban women: an analysis of demographic and health surveys data from 24 African countries, 1991–2014. BMJ Open. 2017 Oct; 7(10):e017344. doi: 10.1136/bmjopen-2017-017344 . 29079606 PMC5665233

[32] The Global Health Observatory. Noncommunicable Diseases: Risk factors 2022[Internet].[cited 12 Dec 2023]. https://www.who.int/data/gho/data/themes/topics/topic-details/GHO/ncd-risk-factors.

[33] World Health Organization. Hypertension [Fact sheet] 2021 [Internet].[cited 12 December 2023]. https://www.who.int/news-room/fact-sheets/detail/ hypertension.

[34] Bivigou EA, Allognon MC, Ndoume F, Mipinda JB, Nzengue EE. Létalité de l’insuffisance cardiaque au Centre Hospitalier Universitaire de Libreville (CHUL) et facteurs associés. Pan African Medical Journal. 2018 Sep; 31(1). doi: 10.11604/pamj.2018.31.27.13259 PMC6430941 30918554

[35] Al-Mawali A, Jayapal SK, Morsi M, Al-Shekaili W, Pinto AD, Al-Kharusi H, et al. Prevalence of risk factors of non-communicable diseases in the Sultanate of Oman: STEPS survey 2017. PLoS One. 2021 Oct; 16(10):e0259239. doi: 10.1371/journal.pone.0259239 . 34710161 PMC8553065

[36] Chiang PP, Lamoureux EL, Shankar A, Tai ES, Wong TY, Sabanayagam C. Cardio-metabolic risk factors and prehypertension in persons without diabetes, hypertension, and cardiovascular disease. BMC Public Health. 2013 Aug 7;13:730. doi: 10.1186/1471-2458-13-730 . 23919264 PMC3751051

[37] Bigna JJ, Nansseu JR, Katte J-C, Noubiap JJ. Prevalence of prediabetes and diabetes mellitus among adults residing in Cameroon: a systematic review and meta-analysis. Diabetes Res Clin Pract. 2018 Mar; 137:109–18. doi: 10.1016/j.diabres.2017.12.005 . 29325776

[38] Millogo T, Bicaba BW, Soubeiga JK, Dabiré E, Médah I, Kouanda S. Diabetes and abnormal glucose regulation in the adult population of Burkina Faso: prevalence and predictors. BMC Public Health. 2018 Mar; 18(1):1–10. doi: 10.1186/s12889-018-5257-4 . 29534705 PMC5851249

[39] Danaei G, Finucane MM, Lu Y, Singh GM, Cowan MJ, Paciorek CJ, et al. National, regional, and global trends in fasting plasma glucose and diabetes prevalence since 1980: systematic analysis of health examination surveys and epidemiological studies with 370 country-years and 27 million participants. Lancet. 2011 Jul; 378(9785):31–40. doi: 10.1016/S0140-6736(11)60679-X . 21705069

[40] Gebreyes YF, Goshu DY, Geletew TK, Argefa TG, Zemedu TG, Lemu KA, et al. Prevalence of high bloodpressure, hyperglycemia, dyslipidemia, metabolic syndrome and their determinants in Ethiopia: Evidences from the National NCDs STEPS Survey, 2015. PloS one. 2018 May;13(5):e0194819. doi: 10.1371/journal.pone.0194819 . 29742131 PMC5942803

[41] Njelekela M, Kuga S, Nara Y, Ntogwisangu J, Masesa Z, Mashalla Y, et al. Prevalence of obesity and dyslipidemia in middle-aged men and women in Tanzania, Africa: relationship with resting energy expenditure and dietary factors. J Nutr Sci Vitaminol 2002 Oct; 48(5):352–8. doi: 10.3177/jnsv.48.352 . 12656207

[42] Faijer-Westerink HJ, Kengne AP, Meeks KA, Agyemang C. Prevalence of metabolic syndrome in sub-Saharan Africa: A systematic review and meta-analysis. Nutr Metab Cardiovasc Dis. 2020 Apr; 30(4):547–65. doi: 10.1016/j.numecd.2019.12.012 . 32143896

[43] Wekesah FM, Nyanjau L, Kibachio J, Mutua MK, Mohamed SF, Grobbee DE, et al. Individual and household level factors associated with presence of multiple non-communicable disease risk factors in Kenyan adults. BMC Public Health. 2018 Nov; 18(3):1–11. doi: 10.1186/s12889-018-6055-8 . 30400905 PMC6219015

[44] Lowe LP, Greenland P, Ruth KJ, Dyer AR, Stamler R, Stamler J. Impact of major cardiovascular disease risk factors, particularly in combination, on 22-year mortality in women and men. Arch Intern Med. 1998 Oct; 158(18):2007–14. doi: 10.1001/archinte.158.18.2007 . 9778200

[45] Yusuf HR, Giles WH, Croft JB, Anda RF, Casper ML. Impact of multiple risk factor profiles on determining cardiovascular disease risk. Prev Med.1998 Jan-Feb; 27(1):1–9. doi: 10.1006/pmed.1997.0268 . 9465349

