# Supplementary material for: Prevalence of cardiometabolic risk factors according to urbanization level, gender and age, in apparently healthy adults living in Gabon, Central Africa
Source: PLoS One. 2024 Apr 5;19(4):e0285907. doi: 10.1371/journal.pone.0285907 (PMC10997135; doi:10.1371/journal.pone.0285907)
Supplement: S1 Table — p-valuea:Men-Women comparison in urban areas; P-valueb:Men-Women comparison in rural areas. (DOCX) [file pone.0285907.s001.docx]

**S1 Table:** **Relationship between the presence of behavioral CMRF and gender**

| Risk factors | Urban (n = 499) | |  | Rural (n = 479) | |  |
| --- | --- | --- | --- | --- | --- | --- |
|  | Men | Women | ***p-value*^a^** | Men | Women | ***p-value*^b^** |
| TOBACCO CONSUMPTION n(%) |  |  |  |  |  |  |
| Tobacco smoking | 20 (11.6) | 11 (3.4) | **< 0.001** | 103 (44.2) | 22 (8.9) | **< 0.001** |
| Daily tobacco smoking during previous 12 months | 11 (6.4) | 03 (1.0) | **< 0.001** | 84 (36.0) | 08 (3.3) | **< 0.001** |
| Occasional tobacco smoking | 09 (5.2) | 08 (2.5) | **0.020** | 19 (8.2) | 14 (5.9) | 0.163 |
| Passive tobacco smoking during previous 30 days | 44 (25.6) | 55 (16.8) | **< 0.001** | 61 (26.2) | 80 (32.5) | **0.027** |
| ALCOHOL CONSUMPTION |  |  |  |  |  |  |
| during previous 12 months | 123 (71.5) | 206 (63.0) | **0.003** | 171 (73.4) | 140 (56.9) | **< 0.001** |
| Excessive consumption during past 7 days | 30 (17.5) | 18 (5.5) | **< 0.001** | 70 (30.0) | 23 (9.4) | **< 0.001** |
| FRUITS AND VEGETABLES INTAKE |  |  |  |  |  |  |
| Number of fruits/days(m±SD) | 1.20 ± 1.3 | 1.31 ± 1.3 | **< 0.001** | 1.30 ± 1.2 | 1.23 ± 1.0 | 0.108 |
| Number of vegetables/day | 1.28 ± 0.9 | 1.41 ± 0.8 | **< 0.001** | 1.40 ± 0.8 | 1.59 ± 0.8 | 0.544 |
| Insufficient fruits/vegetables intake n(%) | 150 (87.2) | 282 (86.2) | 0.641 | 206 (88.4) | 221 (89.8) | 0.469 |
| SALT CONSUMPTION n(%) |  |  |  |  |  |  |
| Always/often adds additional salt during meals | 30 (17.4) | 61 (18.7) | 0.621 | 36 (15.5) | 37 (15.0) | 0.857 |
| Usual consumption of salty additives in food | 35 (20.3) | 86 (26.3) | **0.024** | 101 (43.3) | 98 (39.8) | 0.294 |
| PHYSICAL ACTIVITY n(%) |  |  |  |  |  |  |
| Low | 41 (23.8) | 106 (32.4) | **0.002** | 35 (15.0) | 43 (17.5) | 0.288 |
| Sedentary lifestyle | 09 (5.2) | 36 (11.0) | **< 0.001** | 13 (5.6) | 30 (12.2) | **< 0.001** |

*p-value*^a^:Men-Women comparison in urban areas; *p-value*^b^:Men-Women comparison in rural areas
