# Supplementary material for: Prevalence of cardiometabolic risk factors according to urbanization level, gender and age, in apparently healthy adults living in Gabon, Central Africa
Source: PLoS One. 2024 Apr 5;19(4):e0285907. doi: 10.1371/journal.pone.0285907 (PMC10997135; doi:10.1371/journal.pone.0285907)
Supplement: S2 Table — AOR adjusted for age; P-valuea:Men-Women comparison in urban areas; P-valueb:Men-Women comparison in rural areas. (DOCX) [file pone.0285907.s002.docx]

**S2 Table:** **Multivariate analysis of behavioral risk factors according to gender**

| Variables | Crude OR (95%CI) | Adjusted OR (95%CI) | *p-value*^a^ | Crude OR (95%CI) | Adjusted OR (95%CI) | *p-value*^b^ |
| --- | --- | --- | --- | --- | --- | --- |
|  | Urban area | |  | Rural area | |  |
| Tobacco smoking |  |  |  |  |  |  |
| Men | 3.78 (1.76 - 8.08) | 3.9 (1.82 - 8.39) | < 0.001 | 8.0 (4.85 - 13.41) | 8.0 (4.86 - 13.45) | **< 0.001** |
| Women | Ref | Ref |  |  | Ref |  |
| Excessive alcohol consumption |  |  |  |  |  |  |
| Men | 3.62 (1.96 - 6.72) | 3.7 (1.99 - 6.89) | < 0.001 | 4.1 (2.49 - 6.95) | 4.1 (2.49 - 6.95) | **< 0.001** |
| Women | Ref | Ref |  | Ref | Ref |  |
| Low physical activity |  |  |  |  |  |  |
| Men | Ref | Ref |  | Ref | Ref |  |
| Women | 1.7 (1.1 - 2.61) | 1.83 (1.8 - 2.83) | 0.006 | 1.31 (0.80 - 2.14) | 1.33 (0.81 - 2.18) | 0.254 |
| Sedentary lifestyle |  |  |  |  |  |  |
| Men | Ref |  |  |  |  |  |
| Women | 2.63 (1.2 - 5.67) | 3.04 (1.39 - 6.65) | 0.005 | 2.46 (1.24 - 4.88) | 2.57 (1.28 - 5.15) | **0.007** |

AOR adjusted for age; *p-value*^a^:Men-Women comparison in urban areas; *p-value^b^*:Men-Women comparison in rural areas
