# Supplementary material for: Prevalence of cardiometabolic risk factors according to urbanization level, gender and age, in apparently healthy adults living in Gabon, Central Africa
Source: PLoS One. 2024 Apr 5;19(4):e0285907. doi: 10.1371/journal.pone.0285907 (PMC10997135; doi:10.1371/journal.pone.0285907)
Supplement: S3 Table — P-valuea:Men-Women comparison in urban areas; P-valueb:Men-Women comparison in rural areas; HBP: High blood pressure; LDL-C: Low density lipoprotein-cholesterol; HDL-C: High density lipoprotein-cholesterol; Total-C: Total cholesterol. (DOCX) [file pone.0285907.s003.docx]

**S3 Table:** **Relationship between anthropometric, biological, and number of CMRF with gender**

| Risk factors | Urban (n = 499) | |  | Rural (n = 479) | |  |
| --- | --- | --- | --- | --- | --- | --- |
|  | Men | Women | ***p-value*^a^** | Men | Women | ***p-value*^b^** |
| HBP n(%) | 109 (63.4) | 188 (57.5) | 0.060 | 101 (43.3) | 88 (35.8) | **0.017** |
| Pre-hypertension | 22 (12.8) | 08 (2.4) | **< 0.001** | 37 (15.9) | 32 (13.0) | 0.197 |
| Elevated heart rate | 25 (14.5) | 100 (30.6) | **< 0.001** | 82 (35.2) | 138 (56.1) | **< 0.001** |
| Diabetes | 22 (12.8) | 29 (8.9) | **0.041** | 00 (0.0) | 02 (0.8) | 0.132 |
| Abdominal obesity | 23 (13.4) | 148 (45.3) | **< 0.001** | 09 (3.9) | 108 (43.9) | **< 0.001** |
| Overweight | 39 (22.7) | 93 (28.4) | **0.035** | 51 (21.9) | 73 (29.7) | **0.006** |
| Obesity | 29 (16.9) | 113 (34.6) | **< 0.001** | 17 (7.3) | 74 (30.1) | **< 0.001** |
| Low HDL-C level | 21 (13.6) | 37 (12.2) | 0.554 | 47 (47.0) | 58 (39.5) | 0.102 |
| High Triglycerides level | 03 (1.9) | 06 (2.0) | 1.000 | 05 (5.0) | 00 (0.0) | **--** |
| High LDL-C level | 17 (11.0) | 27 (8.9) | 0.320 | 00 (0.0) | 01 (0.7) | -- |
| High Total-C | 14 (9.1) | 31 (10.2) | 0.576 | 00 (0.0) | 00 (0.0) | -- |
| Hyperglycemia | 38 (22.1) | 75 (22.9) | 0.761 | 43 (18.5) | 41 (16.7) | 0.445 |
| Metabolic Syndrome | 17 (9.9) | 66 (20.2) | **< 0.001** | 08 (3.4) | 31 (12.6) | **< 0.001** |
| NUMBER OF FACTORS |  |  |  |  |  |  |
| None | 01 (0.6) | 02 (0.6) | 0.682 | 00 (0.0) | 03 (1.2) | **0.040** |
| 1 | 18 (10.5) | 35 (10.7) | 0.917 | 18 (7.7) | 26 (10.6) | 0.117 |
| 2 | 37 (21.5) | 65 (19.9) | 0.531 | 51 (21.9) | 45 (18.3) | 0.170 |
| 3 | 42 (24.4) | 78 (23.9) | 0.824 | 85 (36.5) | 70 (28.5) | **0.008** |
| 4 | 46 (26.7) | 81 (24.8) | 0.514 | 54 (23.2) | 72 (29.3) | **0.033** |
| 5 | 20 (11.6) | 49 (15.0) | 0.113 | 19 (8.2) | 24 (9.8) | 0.365 |
| More than 5 | 8 (4.7) | 17 (5.2) | 0.660 | 06 (2.6) | 06 (2.4) | 0.832 |

*p-value*^a^:Men-Women comparison in urban areas*; p-value*^b^:Men-Women comparison in rural areas; HBP: High blood pressure; LDL-C: Low density lipoprotein-cholesterol; HDL-C: High density lipoprotein-cholesterol; Total-C: Total cholesterol.
