# Supplementary material for: Prevalence of cardiometabolic risk factors according to urbanization level, gender and age, in apparently healthy adults living in Gabon, Central Africa
Source: PLoS One. 2024 Apr 5;19(4):e0285907. doi: 10.1371/journal.pone.0285907 (PMC10997135; doi:10.1371/journal.pone.0285907)
Supplement: S4 Table — AOR adjusted for age; P-valuea:Men-Women comparison in urban areas; P-valueb:Men-Women comparison in rural areas; Ref: Reference; HBP: High blood pressure. (DOCX) [file pone.0285907.s004.docx]

**S4 Table:** **Multivariate analysis of biological CMRF according to gender**

| Variables | Crude OR (95%CI) | Adjusted OR (95%CI) | p-value^a^ | Crude OR (95%CI) | Adjusted OR (95%CI) | p-value^b^ |
| --- | --- | --- | --- | --- | --- | --- |
|  | Urban area | |  | Rural area | |  |
| HBP |  |  |  |  |  |  |
| Men | 1.8 (1.21 - 2.82) | 1.6 (1.04 - 2.68) | **0.031** | 1.5 (1.03 - 2.25) | 1.5 (1.03 - 2.30) | **0.032** |
| Women | Ref | Ref |  | Ref | Ref |  |
| Pre-hypertension |  |  |  |  |  |  |
| Men | 8.5 (3.63 - 21.22) | 8.7 (3,61 - 21.12) | **< 0.001** | 1.5 (0.88 - 2.63) | 1.5 (0.88 - 2.63) | 0.124 |
| Women | Ref | Ref |  | Ref | Ref |  |
| Diabetes |  |  |  |  |  |  |
| Men | 1.5 (0.83 - 2.71) | 1.30 (0.68 - 2.49) | 0.421 | - | - |  |
| Women | Ref | Ref |  |  |  |  |
| Metabolic syndrome |  |  |  |  |  |  |
| Men | Ref | Ref |  | Ref | Ref |  |
| Women | 2.3 (1.30 - 4.07) | 2.82 (1.55 - 5.12) | **0.0006** | 4.0 (1.82 - 9.0) | 4.1 (1.83 - 9.14) | **0.001** |
| Overweight |  |  |  |  |  |  |
| Men | Ref | Ref |  | Ref | Ref |  |
| Women | 2.0 (1.29 - 3.23) | 2.3 (1.44 - 3.69) | **< 0.001** | 2.38 (1.54 - 3.68) | 2.5 (1.60 - 3.90) | **< 0.001** |
| Obesity |  |  |  |  |  |  |
| Men | Ref | Ref |  | Ref | Ref |  |
| Women | 3.34 (2.0 - 5.43) | 4.2 (3.54 - 7.14) | **< 0.001** | 7.25 (4.05 - 12.99) | 7.6 (4.22 - 13.74) | **< 0.001** |
| Abdominal obesity |  |  |  |  |  |  |
| Men | Ref | Ref |  | Ref | Ref |  |
| Women | 5.35 (3.26 - 8.74) | 7.2 (4.29 - 12.32) | **< 0.001** | 19.5 (9.55 - 39.7) | 20.4 (9.98 - 41.94) | **< 0.001** |

AOR adjusted for age; p-value^a^:Men-Women comparison in urban areas; p-value^b^:Men-Women comparison in rural areas; Ref: Reference; HBP: High blood pressure.
